# Hepatitis E Virus in Pork Liver Sausage, France

**DOI:** 10.3201/eid1902.121255

**Published:** 2013-02

**Authors:** Alessandra Berto, Sylvia Grierson, Renate Hakze-van der Honing, Francesca Martelli, Reimar Johne, Jochen Reetz, Rainer G. Ulrich, Nicole Pavio, Wim H.M. Van der Poel, Malcolm Banks

**Affiliations:** Author affiliations: Animal Health and Veterinary Laboratories Agency, Weybridge, UK (A. Berto, S. Grierson, F. Martelli, M. Banks);; Wageningen University and Research Centre Central Veterinary Institute, Lelystad, the Netherlands (A. Berto, R. Hakze-van der Honing, W.H.M. Van der Poel);; Federal Institute for Risk Assessment, Berlin, Germany (R. Johne, J. Reetz);; Friedrich-Loeffler-Institut Institute for Novel and Emerging Infectious Diseases, Greifswald-Insel Riems, Germany (R.G. Ulrich);; French Agency for Food, Environmental and Occupational Health and Safety, Paris, France (N. Pavio); ‘; University of Liverpool National Consortium for Zoonosis Research, Liverpool, UK (W.H.M. Van der Poel)

**Keywords:** Hepatitis E, hepatitis E virus, foodborne disease, pork, food chain, 3D cell culture, viruses, France, HEV, sausage, pork liver, pig liver, contamination

## Abstract

We investigated viability of hepatitis E virus (HEV) identified in contaminated pork liver sausages obtained from France. HEV replication was demonstrated in 1 of 4 samples by using a 3-dimensional cell culture system. The risk for human infection with HEV by consumption of these sausages should be considered to be high.

Foodborne transmission of hepatitis E virus (HEV) to humans from consumption of undercooked pig liver and deer meat has been reported in Japan (*1*–*3*). In addition, commercial pig livers purchased from local grocery stores in Japan, the United States, and Europe were contaminated with HEV (*4*–*6*). Epidemiologic and PCR results linked a cluster of autochthonous acute hepatitis E cases to ingestion of raw figatelli, which is a dried, cold-smoked sausage containing ≈30% pig liver (*7*). We investigated the viability of HEV in pork liver sausages produced in France.

## The Study

Four samples of pork liver sausage (designated sausages A–D) collected at the final production stage from 4 independent manufacturers in 3 locations in southern France were found to be HEV positive by using real-time reverse transcription PCR (RT-PCR). Samples were tested in 2 institutes, the Animal Health and Veterinary Laboratories Agency, United Kingdom, and Wageningen University and Research Centre, the Netherlands. Three-dimensional (3D) cell culture propagation was performed to investigate the presence of infectious HEV particles. PLC/PRF/5 hepatocarcinoma cells (American Type Culture Collection 8024) were cultured as a monolayer (2D) at 37°C in GTSF-2 medium (8) in a 5% CO_2_ environment. Cells were trypsinized at 95% confluence and resuspended in fresh medium to a density of 2 × 10^5^ cells/mL. Fifty milliliters of cell suspension was then introduced into a rotating wall vessel with 10 mg/mL of porous Cytodex-3 microcarrier beads (collagen type I–coated porous microspheres, average diameter 175 μm (Sigma, Dorset, UK and Zwijndrecht, the Netherlands) and incubated at 37°C in 5% CO_2_. The cells were incubated for >28 d before inoculation to enable complete differentiation.

The inoculum was prepared by homogenizing a 2.5-mg fragment of each sample with mortar and pestle in 5 mL of culture medium. The homogenate was centrifuged at 8,000 × *g* for 3 min, and the supernatant was filtered sequentially through 1.2-µm, 0.45-µm, and 0.2-µm filters to reduce the risk for bacterial contamination. The medium was removed from the rotating wall vessel, and 2.5 mL of inoculum was incubated with the cells for 2 h at 35.5°C; at that point, 47.5 mL of fresh medium was added. Subsamples of medium (140 µL) were collected in duplicate, added to 560 µL of lysis buffer (QIAamp Viral RNA Mini Kit; QIAGEN, Crawley, UK, and Venlo, the Netherlands), and stored at −20°C until RNA extraction was performed.

Both institutes performed real-time RT-PCR by using primers and probe as described (*9*), using the Superscript III Platinum One-Step Quantitative RT-PCR System (Invitrogen, Paisley, UK, and Bleiswijk, the Netherlands). Negative (water) and positive (extract from positive fecal sample, genotype 3) controls were included. 

**Figure 1:**
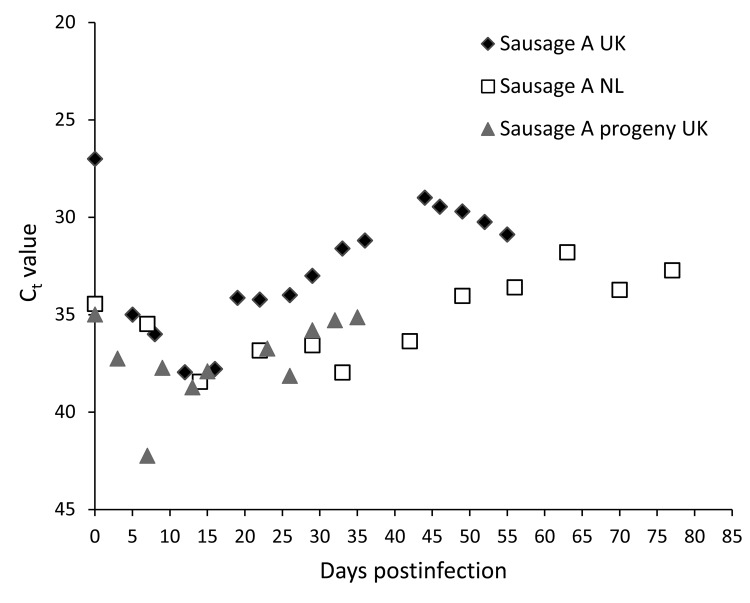
Cycle threshold (C_t_) values detected by real-time reverse transcription PCR for hepatitis E virus–positive supernatant of 3D cells infected with homogenate of pork sausages from France. Diamonds indicate testing of sausage A in the United Kingdom; squares indicate testing of sausage A in the Netherlands; triangles indicate testing of progeny of sausage A in the United Kingdom.

HEV RNA was detected in the 3D cell culture supernatants of all 4 sausage samples up to 8 d postinfection (dpi). Thereafter, HEV RNA was detected only in the cells inoculated with the sample A homogenate (Figure 1); it was assumed that the signals from the other 3 samples represented residual inoculum or an abortive infection. The sample A culture showed a cycle threshold (C_t_) value of 27 on the day of inoculation (dpi 0) that increased to 35 on dpi 5, continued to increase until dpi 11, when it peaked at 38 and then began to decrease. A low C_t_ value of 29 was observed on dpi 44. Similar results were obtained in the laboratory in the Netherlands (Figure 1).

To evaluate the infectivity of progeny viruses from the primary inoculation, supernatant positive for HEV by RT-PCR from dpi 16 was used to infect fresh 3D PLC/PRF/5 cultures according to the protocol described for the primary inoculation. Cells infected with the supernatant from the original sample A homogenate cultures had positive HEV RNA test results on most days after inoculation; C_t_ values remained relatively constant at an average of 37 from immediately after inoculation (dpi 0) to the end of the experiment at dpi 35.

To compare the cultured virus to the inoculum, a partial fragment of the open reading frame 2 of HEV extracted from culture subsamples (dpi 16, dpi 55; progeny subsample at dpi 35) was sequenced as described (*10*). HEV RNA sequences detected in the culture subsamples were characterized and demonstrated 100% identity with that of the inoculum (304 bp of open reading frame 2) but differed from the control strain used. These sequences were confirmed as HEV genotype 3.

**Figure 2:**
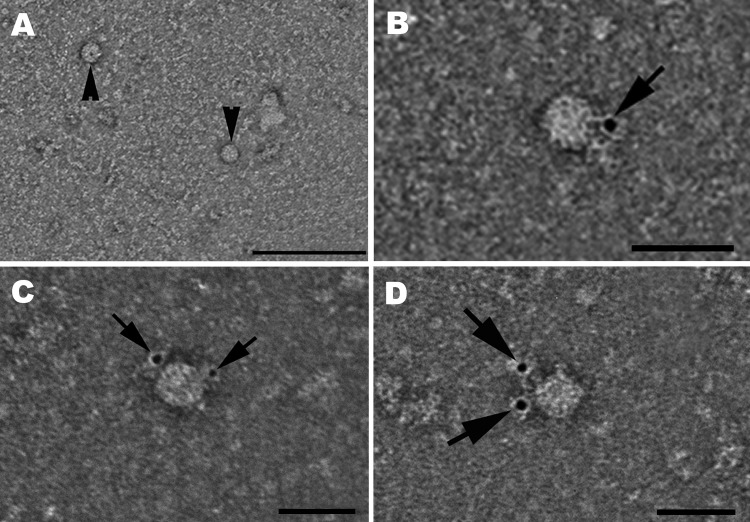
Hepatitis E virus (HEV) particles in the cell culture supernatant of pork liver sausage sample A, collected at 33 dpi. A) Transmission electron micrograph of negatively stained HEV particles ≈33 and 34 nm (arrowheads). Scale bar indicates 200 nm. B–D) Hepatitis E virions ≈28 (B), 33 (C), or 32 (D) nm in diameter, identified by using an HEV genotype 3–specific rabbit hyperimmune serum and a gold-labeled secondary antibody. Arrows show bound gold particles. Scale bars indicate 50 nm.

Further confirmation of the presence of viable virus particles in cell culture was sought by using electron microscopy. Supernatants of the cell cultures were applied to Pioloform/carbon-coated, 400-mesh copper grids (Plano GmbH, Wetzlar, Germany) for 10 min, fixed with 2.5% aqueous glutaraldehyde solution for 1 min, and stained with 2% aqueous uranyl acetate solution for 1 min. The specimens were examined through transmission electron microscopy by using a JEM-1010 microscope (JEOL, Tokyo, Japan) at an 80-kV accelerated voltage. HEV particles were observed in the supernatant of the sample A culture collected at dpi 33 (Figure 2, panel A). For additional proof by immunoelectron microscopy, an *Escherichia coli*–expressed, His-tagged HEV genotype 3 capsid protein derivative harboring amino acid residues 326–608 (*11*) was used to generate HEV-positive serum in a rabbit by 3 subcutaneous inoculations at 4-week intervals (P. Dremsek et al., unpub. data). The immunoelectron microscopy examination of this serum and goat anti-rabbit IgG linked with 5-nm gold particles (BBInternational, Oconomowoc, WI, USA) confirmed the presence of HEV particles (Figure 2, panels B–D).

## Conclusions

We confirmed that 1 sample of pork liver sausage that had positive test results for HEV RNA by real-time RT-PCR contained viable HEV. We cultured 4 sausage samples in 2 different institutes by performing 3D cell-culture propagation of HEV; HEV replication was detected in the same sample independently in both institutes, and the replicated virus was shown to be infectious. We observed entire, cell-free virus particles by transmission electron microscopy at dpi 33, providing further proof of in vitro replication of the virus that contaminated the pork liver sausage. 

We conclude that pork liver sausages can contain infectious HEV and that consuming these products should be regarded as a risk factor for HEV infection. Furthermore, we have shown the potential of the 3D culture system to correlate the presence of HEV RNA with the presence of infectious HEV particles.
